# Supplementary material for: The Evolution and Characterization of the RNA Interference Pathways in Lophotrochozoa
Source: Genome Biol Evol. 2024 May 7;16(5):evae098. doi: 10.1093/gbe/evae098 (PMC11114477; doi:10.1093/gbe/evae098)
Supplement: evae098_Supplementary_Data [file evae098_supplementary_data.zip › Supplementary_materials.zip!/selected_miRNAs/NoGe_selected_miRNAs/known_miRNA_families_pdf_files/NMRB01004296.1_Notospermus_geniculatus_strain_Ushimado_scaffold4296,_whole_genome_shotgun_sequence_45764.pdf]

Provisional ID	:	NMRB01004296.1_Notospermus_geniculatus_strain_Ushimado_scaffold4296,_whole_genome_shotgun_sequence_45764
Score total	:	6183.8
Score for star read(s)	:	3.9
Score for read counts	:	6175.1
Score for mfe	:	1.7
Score for randfold	:	0
Score for cons. seed	:	3
Total read count	:	12124
Mature read count	:	12114
Loop read count	:	0
Star read count	:	10

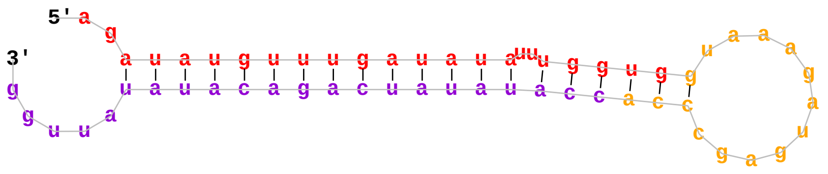

Genome coordinates: NMRB01004296.1 960-1020

pre-miRNA sequence:
AGAUAUGUUUGAUAUAUUUGGUGAGAUAUGUUUGAUAUAUUUGGUGGUAAAGAUGAGCCCACCAUAUAUCAGACAUAUAUUGG

Mature sequence: AGAUAUGUUUGAUAUAUUUGGUG

Aligned mature sequence of the *N. geniculatus* pre-miRNA with the top 5 hits from the BLAST search against the pre-miRNA mirGeneDB set:

```
                                   **************** ***
    Nge_Mir-190                   agauauguuugauauauuuggug-
    Cte-Mir-190_5p                agauauguuugauauauuuggugg
    Efe-Mir-190-P11_5p            agauauguuugauauauuuggugg
    Efe-Mir-190-P12_5p            agauauguuugauauauuuggugg
    Aca-Mir-190-P1_5p             ugauauguuugauauauuagguug
    Cgi-Mir-190_5p                agauauguuugauauauuuggug-
```
